# Synergistic Effect of Doripenem and Cefotaxime to Inhibit CTX-M-15 Type β-Lactamases: Biophysical and Microbiological Views

**DOI:** 10.3389/fphar.2017.00449

**Published:** 2017-07-05

**Authors:** Lubna Maryam, Asad U. Khan

**Affiliations:** Interdisciplinary Biotechnology Unit, Medical Microbiology and Molecular Biology Laboratory, Aligarh Muslim UniversityAligarh, India

**Keywords:** antibiotic resistance, doripenem, UV spectroscopy, fluorescence quenching, CD spectroscopy, catalytic efficiency, fractional inhibitory concentration

## Abstract

CTX-M-15 type β-lactamase has the ability to hydrolyse cefotaxime, a third generation cephalosporin. The infections caused by multidrug resistant strains, especially CTX-M-15 producing strains are being treated with carbapenem group of β-lactam antibiotics. The objective of the study was to know if cefotaxime in combination with doripenem (carbapemen antibiotic) at very low concentration, inhibits the CTX-M-15 producing bacterial strains. *bla*_CTX−M−15_ gene was cloned to express CTX-M-15 enzyme and construct CTX-M-15 producing strain. The clone carrying CTX-M-15 was found susceptible to doripenem. Doripenem and CTX-M-15 binding was an endothermic and spontaneous process leading to change in polarity in the micro-environment and conformational changes of enzyme as shown by fluorescence, UV and CD spectroscopic study. The catalytic efficiency of CTX-M-15 enzyme was reduced to about 15.86% when it was treated with doripenem along with cefotaxime (in 5 times molar ratio each of doripenem and cefotaxime w.r.t CTX-M-15), as compared to the studies where enzyme's efficiency was increased by 33% when treated with cefotaxime alone. Hence, doripenem in combination with cefotaxime reduces enzyme's efficiency to hydrolyse cefotaxime by about 48%. FIC study showed that doripenem paired with cefotaxime showed synergistic effect against CTX-M-15 producing bacterial strain. The study concludes that doripenem at very low concentration (25 nM), induces such a structural changes in CTX-M-15 which reduced enzyme's activity to hydrolyse cefotaxime. Hence, the synergistic use of doripenem and cefotaxime plays a significant role in inhibiting the efficiency of CTX-M-15 type β-lactamase, and may provide an alternative approach to reduce the resistance against the cephalosporin type antibiotics.

## Introduction

Production of β-lactamases is one of the major mechanisms in bacteria to develop resistance against β-lactam antibiotics (Bonnet, 2004). It arises due to the cleavage of amide bond in β-lactam rings of these antibiotics (Bush, 2010), making the problem of resistance, an emerging public health concern worldwide (Sun et al., 2010).

Cephalosporins are a class of β-lactam antibiotics used for the treatment of both positive and negative bacterial infections. These antibiotics act by targeting bacterial penicillin binding proteins (PBPs). Resistance to cephalosporin developed when PBPs are protected by β-lactamases (Livermore, 1987). The production of high level of chromosomal and plasmid mediated β-lactamases by β-lactam producing strains is the major cause of resistance.

One of the class A belonging β-lactamases, CTX-M-15, has the ability to hydrolyse cephalosporins (Coque et al., 2008). It is the first reported enzyme having the capability to hydrolyse cefotaxime (a cephalosporin; Matsumoto et al., 1988). The main mechanism underlying emergence of resistance against cephalosporin group of antibiotics are environmental dissemination of *bla*_CTX−M−15_ gene via mobile elements and their further mutations, leading to appearance of newer variants in the population (Canton et al., 2012). Over the last 10 years almost all extended spectrum β-lactamases are replaced by CTX-M enzymes, this is due to high rate of mobilization of *bla*_CTX−M_ gene to different genetic elements as compared to other class A β-lactamases producing strains (Barlow et al., 2008).

This major public health concern due to emergence of cephalosporin resistance has left us with narrow options to treat bacterial infections. To combat this problem, carbapenems are used to treat such infections (Park, 2014). It is a group of antibiotics which are effective against carbapenemase producing strains of bacteria (Papp-Wallace et al., 2011). Due to their expanded spectra and the ability to avoid development of resistance they are used as last resort antibiotics. Doripenem, one of the antibiotic of carbapenem group is used against CTX-M type β-lactamase producing bacteria because of its broad spectrum coverage and useful clinical outcomes (Mandell, 2009). It has same mechanism of action against β-lactamase producing strains of bacteria as other β-lactam antibiotics. Moreover, doripenem shows higher level of potency in preventing the growth of resistant strains with respect to other carbapenems (Mushtaq et al., 2004; Sakyo et al., 2006).

However, doripenem administration causes common side effects such as headache, nausea, diarrhea, skin problems, lower blood counts, inflammation of the walls of veins, etc. And administration of cefotaxime antibiotic is ineffective since it is resistant to CTX-M-15 producing strains. It is reported that the combination of antibiotic therapy can be effective for treatment even if the bacteria is resistant to individual antibiotics (Tängdén, 2014). Also in comparision to monotherapies, combination therapy is better treatment option since it can overcome broader antibacterial spectrum, reduce the risk of resistance and show synergistic effect. In one of the earlier studies it was observed that the treatment of CTX-M-15 enzyme with cefotaxime and streptomycin when used in combination, decreased the efficiency of enzyme considerably (Maryam and Khan, 2016). Moreover, if any drug is effective at its reduced dose, the issues related to its side effects and overdose can also be prevented.

In view of the above background, we have initiated this study to understand the effect of minimal quantity of doripenem in combination with cefotaxime against CTX-M-15 type β-lactamase producing strain.

## Material

### Source of protein/enzyme

EC-15 clinical strain of *Enterobacter cloacae* (Chen et al., 2014) was used for cloning of CTX-M-15 gene in high expression cloning vector pQE-2 and transformed into competent *E. coli* BL21 (DE3) cells.

### Chemicals and antibiotics

Inducer Isopropyl-β-D-1-thiogalactopyranoside (IPTG) was purchased from Roche (Basel, Switzerland). Imidazole was purchased from Sigma-Aldrich. Doripenem was purchased from Aureate (India). Nitrocefin was purchased from Calbiochem (USA). Chemicals and reagents used were of analytical grade and double distilled water was used during the study.

## Methodology

### Cloning *bla*_CTX−M−15_, expression, and purification of CTX-M-15

*bla*_CTX−M−15_ was cloned from EC-15 clinical strain of *E. cloacae* and protein was expressed and purified to homogeneity as described earlier (Maryam and Khan, 2016, 2017). The concentration and purity of CTX-M-15 protein was determined on a double beam Shimadzu UV–vis spectrophotometer (UV-1800), using a molar extinction coefficient of 25,440 M^−1^ cm^−1^ at 280 nm (Faheem et al., 2013).

### Antibiotic susceptibility testing

MIC of doripenem against BL21 (DE3) cells harboring *bla*_CTX−M−15_ gene was determined by micro dilution method according to Clinical Laboratory Standards Institute (CLSI) laid guidelines (Clinical Laboratory Standards Institute, 2011).

### UV absorption studies

UV absorption study was carried out on Shimadzu UV-1800 double beam spectrophotometer of Shimadzu International Co. Ltd., Kyoto, Japan. Absorption peak obtained was mainly accredited to the absorptions of tryptophan residues which are present in CTX-M-15 (Edelhoch, 1967). Absorption of CTX-M-15 alone and in presence of varying concentrations of doripenem were taken in 250–300 nm wavelength range.

### Fluorescence spectroscopic study

To reveal the mechanism of interaction of doripenem with CTX-M-15 protein (Eftink and Ghiron, 1976; Lakowicz, 1988), at 298, 303, and 308 K, fluorescence spectrometric study was carried out as described previously (Maryam and Khan, 2016). Two micromolars of CTX-M-15 protein in sample of 450 μl was taken and to it 2 μM of doripenem was successively added till total volume reached 30 μl.

### Circular dichroism spectroscopic study

CD spectra measurements were carried out to monitor the effect of doripenem binding on the structure of CTX-M-15 using the method described earlier (Maryam and Khan, 2016). Far and near UV CD spectra of CTX-M-15 were obtained in the absence and in the presence of doripenem in 1, 5, and 10 μM ratios of CTX-M-15 protein. The spectra were recorded in 0.1 and 1.0 cm pathlength cuvettes taking protein concentrations of 5 and 15 μM, respectively. The corrections were made for appropriate blanks.

### Steady-state enzyme kinetics study

To get an insight effect on hydrolytic activity of the CTX-M-15 enzyme toward nitrocefin, a choromogenic cephalosporin substrate (O'Callaghan et al., 1972) in the presence of doripenem, steady state enzyme kinetics study was carried out on Shimadzu UV-1800 double beam spectrophotometer (Shimadzu International Co. Ltd., Kyoto, Japan) by observing the velocities of appearance of nitrocefin and its disappearance. The catalytic activity was monitored at 298 K in 50 mM phosphate buffer at pH 7.4. Five nanomolars of CTX-M-15 enzyme was taken along with addition of 20 μg/ml BSA (Galleni et al., 1994) to the final volume, in order to prevent CTX-M-15 denaturation and for its dilution, since BSA added in this concentration, does not affect the hydrolytic ability of the enzyme. CTX-M-15 activity in the absence of doripenem and in the presence of 5, 25, and 50 nM of doripenem was observed by varying the concentration of nitrocefin from 0 to 800 μM. Another enzyme kinetic study was carried out taking 5 nM of CTX-M-15 enzyme at pH 7.4 and temperature 298 K in 50 mM phosphate buffer in the presence of 25 nM of doripenem and 25 nM of cefotaxime. Appearance of red colored nitrocefin as product is determined at 486 nm by measuring its hydrolysis for 70 s and the concentration was obtained by measuring the absorbance by taking the value of molar extinction coefficient 15,000 M^−1^cm^−1^ at 486 nm.

### Checkerboard microdilution assay

2D checkerboard microdilution assay was performed on 96-well microtiter plate to investigate the interaction between doripenem and cefotaxime with BL21 (DE3) cells harboring *bla*_CTX-M-15_ gene. Doripenem and cefotaxime were taken in concentrations below, equal to and above their MICs and serial dilution of the two drugs were carried out. MIC of cefotaxime for *E. coli* DH5α cells harboring *bla*_CTX-M-15_ gene was reported to be 512 μg/ml (Faheem et al., 2013). Fractional inhibitory concentration (FIC) of both doripenem and cefotaxime was calculated to monitor the effect of these two drugs in combination. Using the following equations the FIC index (FICI) was calculated (Hasan et al., 2013).

- FIC of doripenem = (MIC of doripenem in combination)/(MIC of doripenem)
- FIC of cefotaxime = (MIC of cefotaxime in combination)/(MIC of cefotaxime)
- FICI = FIC of doripenem + FIC of cefotaxime                        (11)

## Results

The purity of CTX-M-15 protein was found to be around 94% as observed by a single band of 31 kDa on SDS-PAGE (Figure S1). The final concentration of the protein was calculated as 2.8 mg/ml, taking molar extinction coefficient of 25, 440 M^−1^ cm^−1^, at 280 nm.

The Minimum inhibitory concentration of doripenem for BL21 (DE3) cells harboring *bla*_CTX−M−15_ gene was determined using micro dilution method and it was observed as ≤0.5 mg/L (1189.05 nM) which is in the sensitive range. While, MIC of cefotaxime was found to be 512 mg/L, in resistant range.

**Figure 1:**
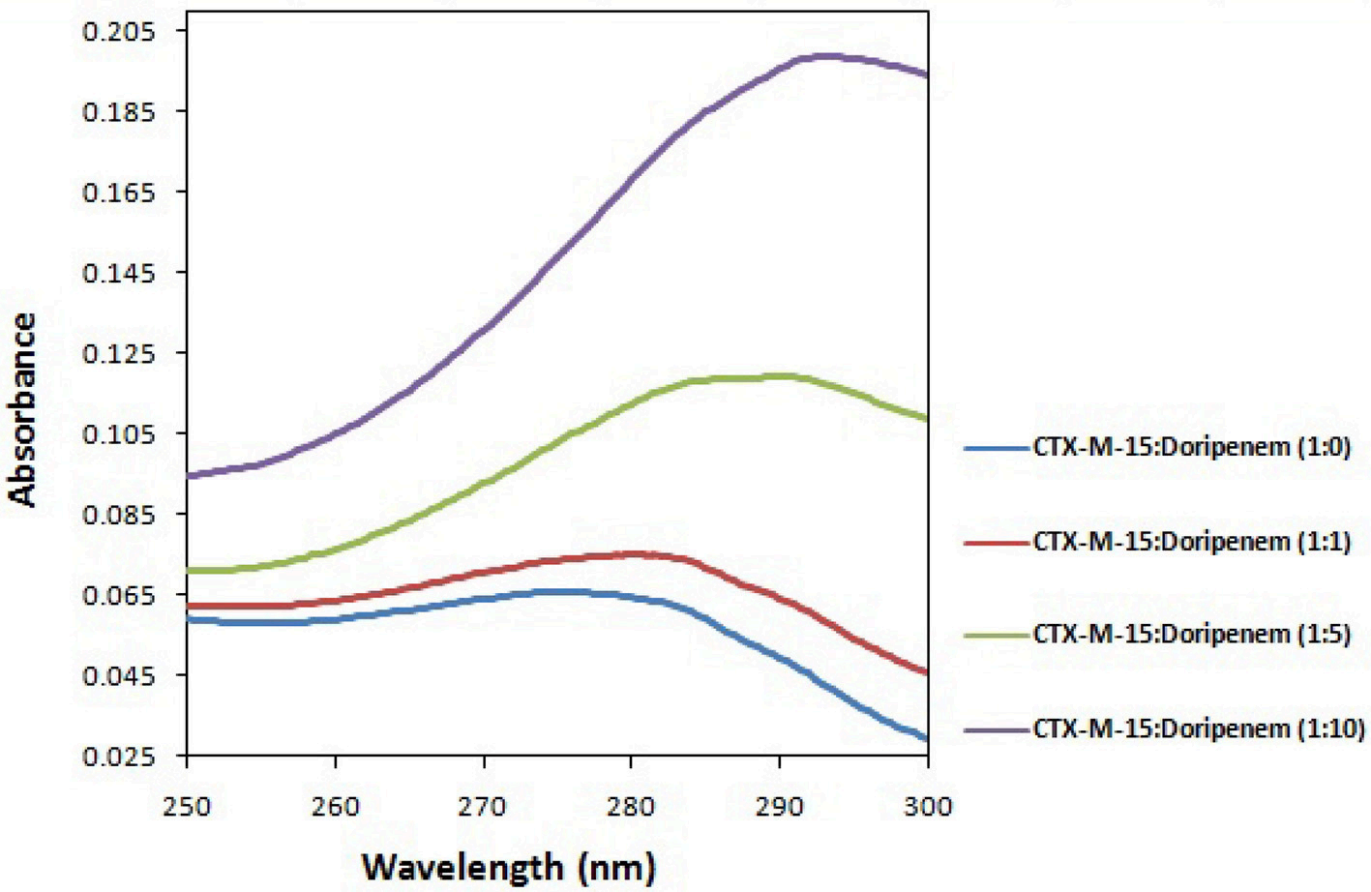
UV absorption spectra of CTX-M-15 (2 μM) between 250 and 300 nm wavelength range in the absence and presence of 2, 10, and 20 μM of doripenem. The spectra was taken at 298 K in 50 mM sodium phosphate buffer of pH 7.4.

UV absorption spectroscopy was carried out to check the structural changes occurred upon protein-drug interaction. The absorption of CTX-M-15 was increased with the increasing concentration of doripenem and the red shift was shown by maximum highest peak of the spectra (Figure 1).

**Figure 2:**
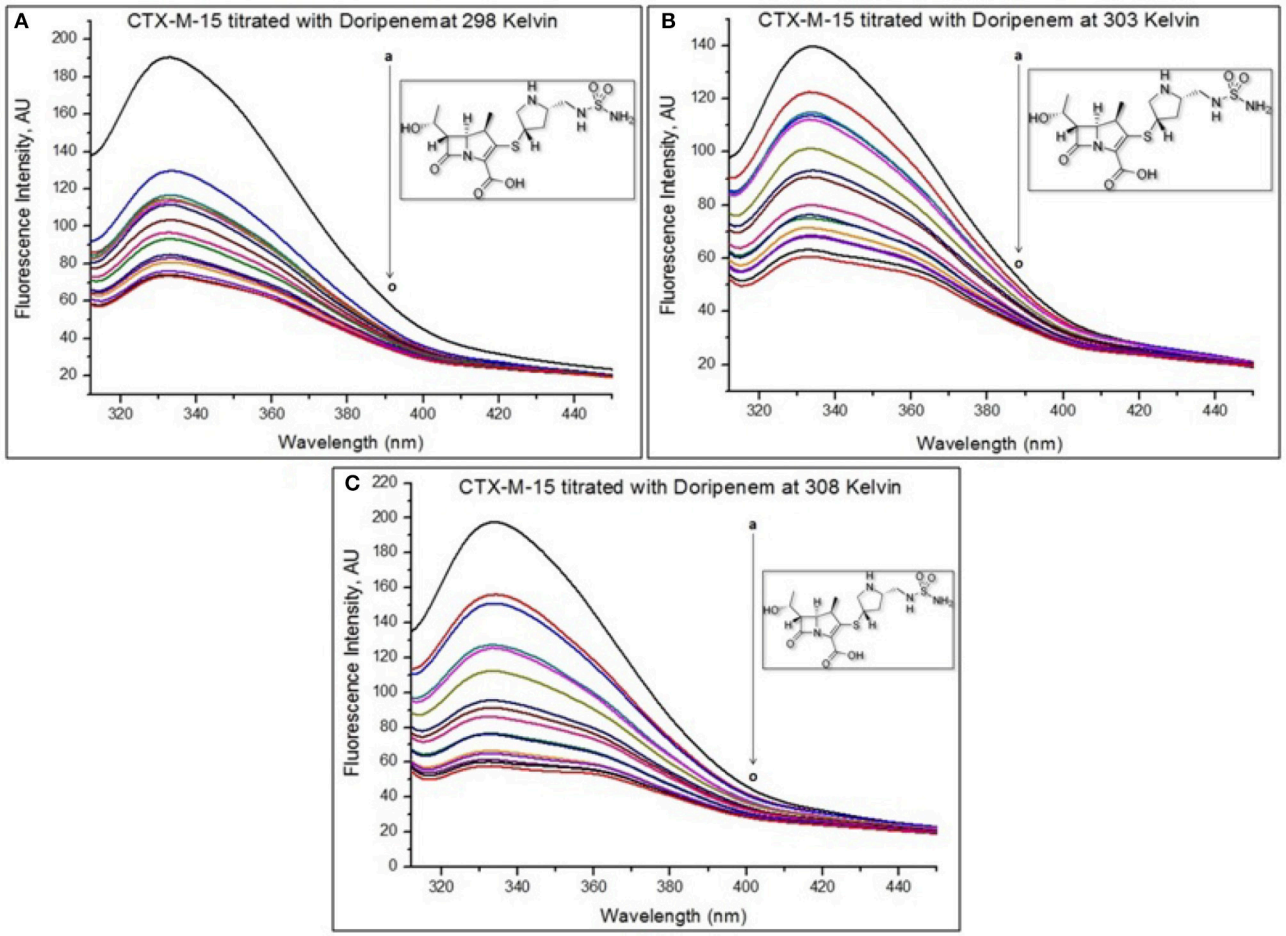
The intrinsic fluorescence of the protein was measured at 298 K **(A)**, 303 K **(B)**, and 308 K **(C)** upon excitation at 295 nm in 50 mM sodium phosphate buffer of pH 7.4. Figure shows doripenem-induced fluorescence quenching of CTX-M-15. The concentration of CTX-M-15 taken was 2 μM, and the concentration of doripenem was varied from 0 to 30 μM (a–o) in a successive increment of 2 μM Inset shows the structure of doripenem.

**Figure 3:**
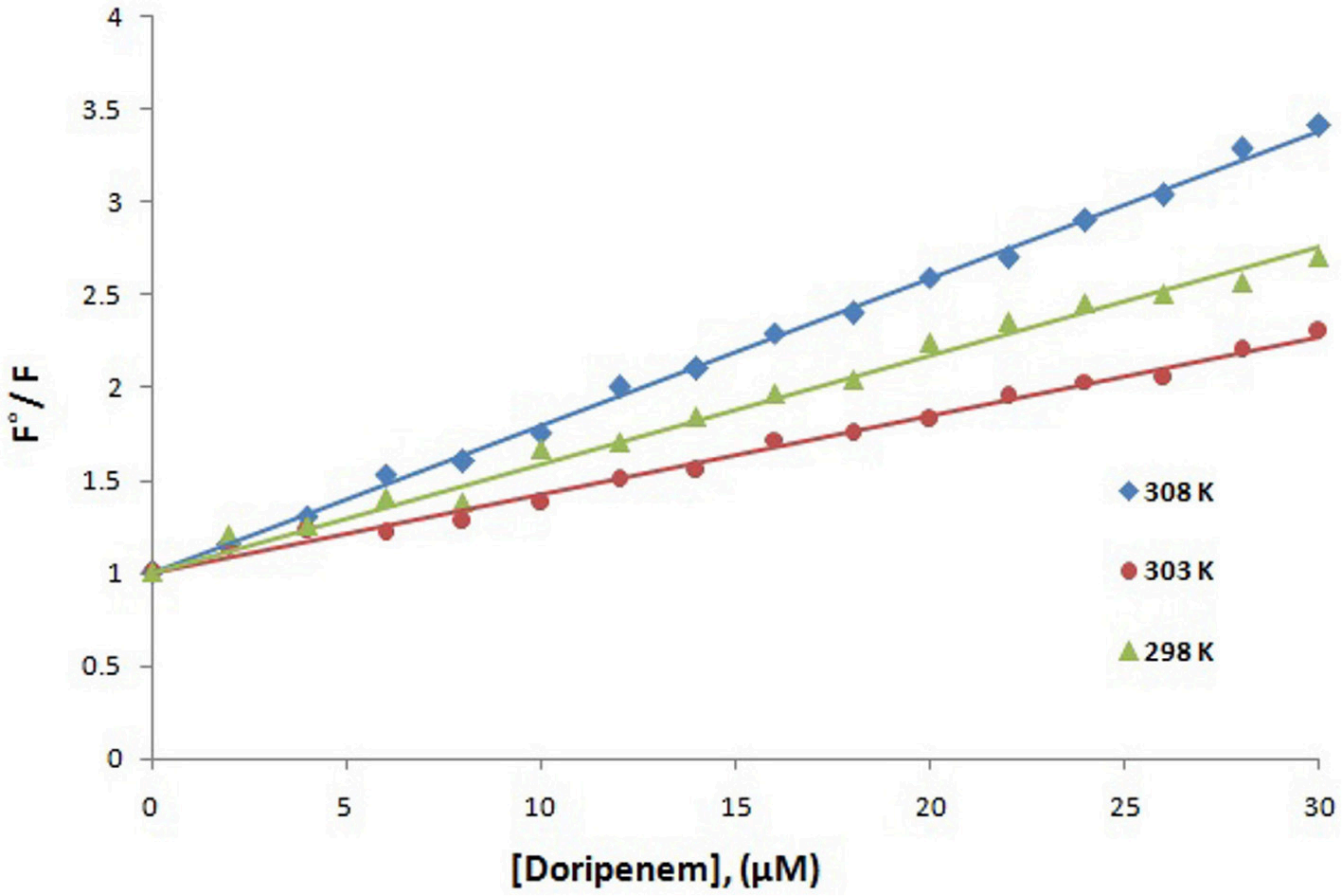
Stern-Volmer plot for quenching of CTX-M-15 (2 μM) in presence of doripenem (from 0 to 30 μM) in 50 mM sodium phosphate buffer at pH 7.4 is shown.

**Figure 4:**
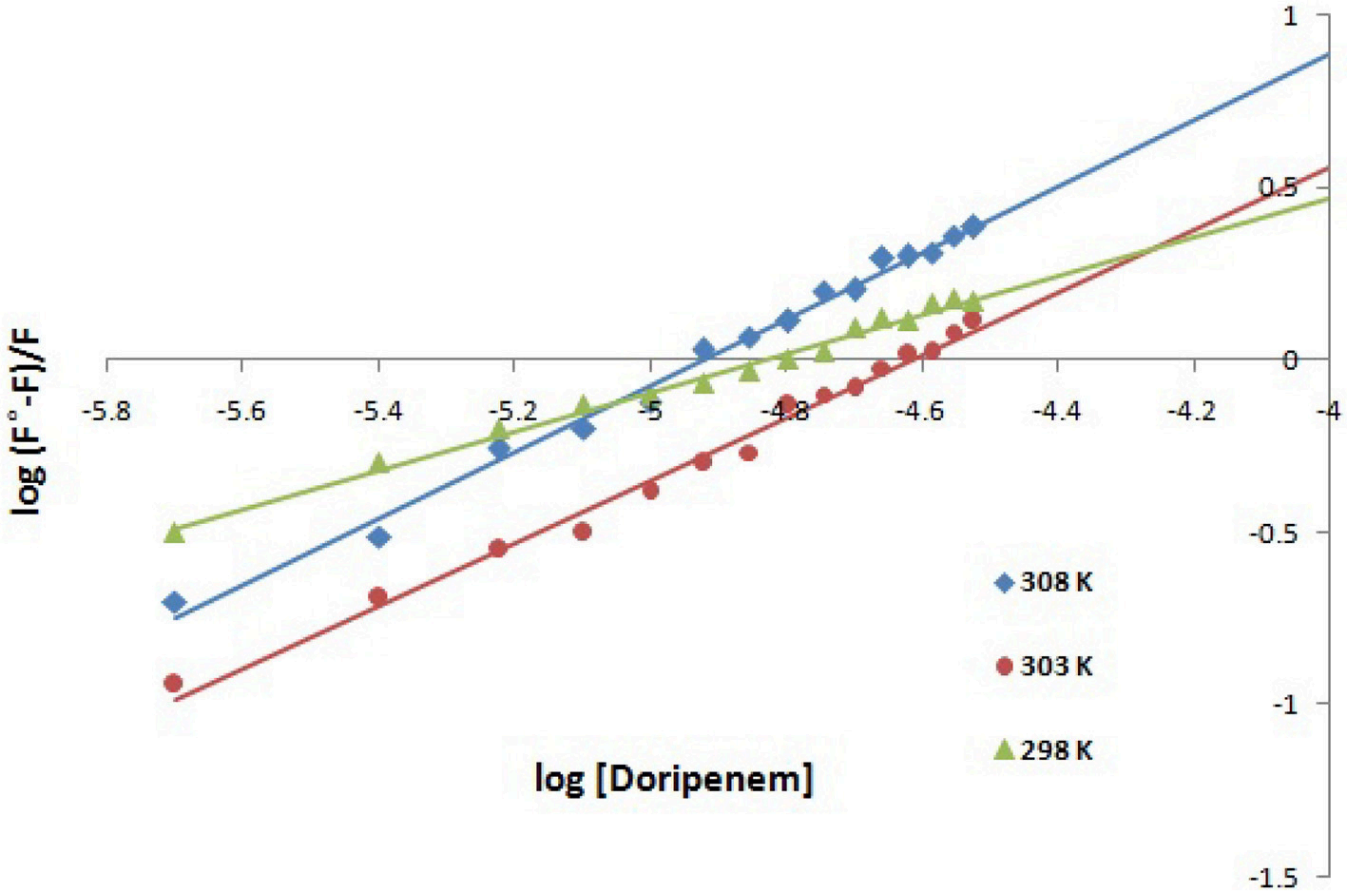
Modified Stern-Volmer plot for quenching of CTX-M-15 (2 μM) in presence of doripenem (from 0 to 30 μM) in 50 mM sodium phosphate buffer at pH 7.4 is shown.

**Figure 5:**
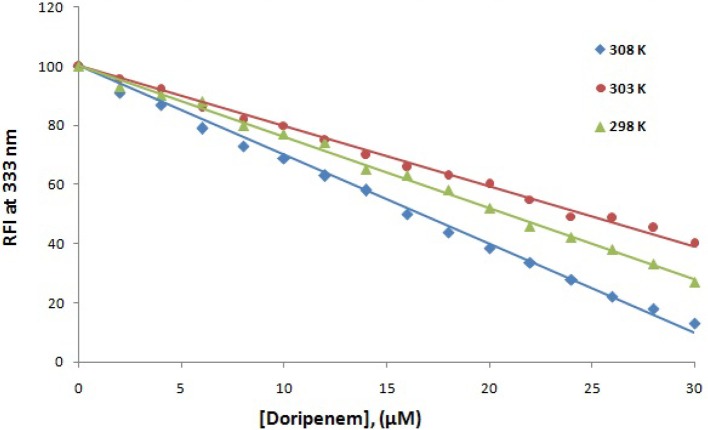
The decrease in relative fluorescence intensity (RFI) of CTX-M-15 (2 μM) in the presence of doripenem (0 to 30 μM ) at 298, 303, and 308 K in 50 mM sodium phosphate buffer at pH 7.4 is shown.

Fluorescence spectroscopy was performed to understand the binding phenomenon of doripenem. A progressive decrease in the fluorescence intensity curve was observed due to quenching of CTX-M-15 during binding of doripenem on CTX-M-15 at three temperatures of 298, 303, and 308 K (Figure 2). Maximum decrease in relative fluorescence intensity was observed at 308 K proceeded by relative fluorescence intensity at 298 and 303 K (Figure 5). Analysis of K_SV_ (Stern–Volmer constant) and [Q] (concentration of quencher in moles) was done using the Stern–Volmer equation (Lakowicz, 1988) (Figure 3, Table 1):

(1) F°/F= 1+Ksv[Q]=1+Kqτ°[Q]

where F is the fluorescence intensity of CTX-M-15 in the absence of doripenem and F is the fluorescence intensity of CTX-M-15 in the presence of (quencher) doripenem and Kq is the bimolecular quenching rate constant which was calculated using the relation:

(2) Kq=Ksv/τ°

where τ° is the mean lifetime of Tryptophan fluorescence of CTX-M-15 protein in the absence of doripenem which is approximately equal to 4.31 × 10^−9^ s. Moreover, using the following modified Stern–Volmer Equation (3) (Figure 4, Table 1), the binding constant (Ka) and number of binding sites (*n*) are calculated (Kang et al., 2004).

(3) $$\text{log}\frac{\text{F}{}^{\circ}-\text{F}}{\text{F}}=\log\text{Ka}+\text{nlog}[\text{Q}]$$

**Table 1:** Stern–Volmer quenching constants and binding parameters for CTX-M-15 and doripenem interaction.

**Temperature (K)**	**Ksv(M^−1^)**	**Kq(M^−1^s^−1^)**	**Ka(M^−1^)**	***n***	***R*^2^**
298	5.8 × 10^4^	1.3 × 10^13^	1.35 × 10^2^	0.44	0.990
303	4.2 × 10^4^	0.97 × 10^13^	1.31 × 10^4^	0.894	0.990
308	8.2 × 10^4^	1.9 × 10^13^	3.13 × 10^4^	0.911	0.993

**Figure 6:**
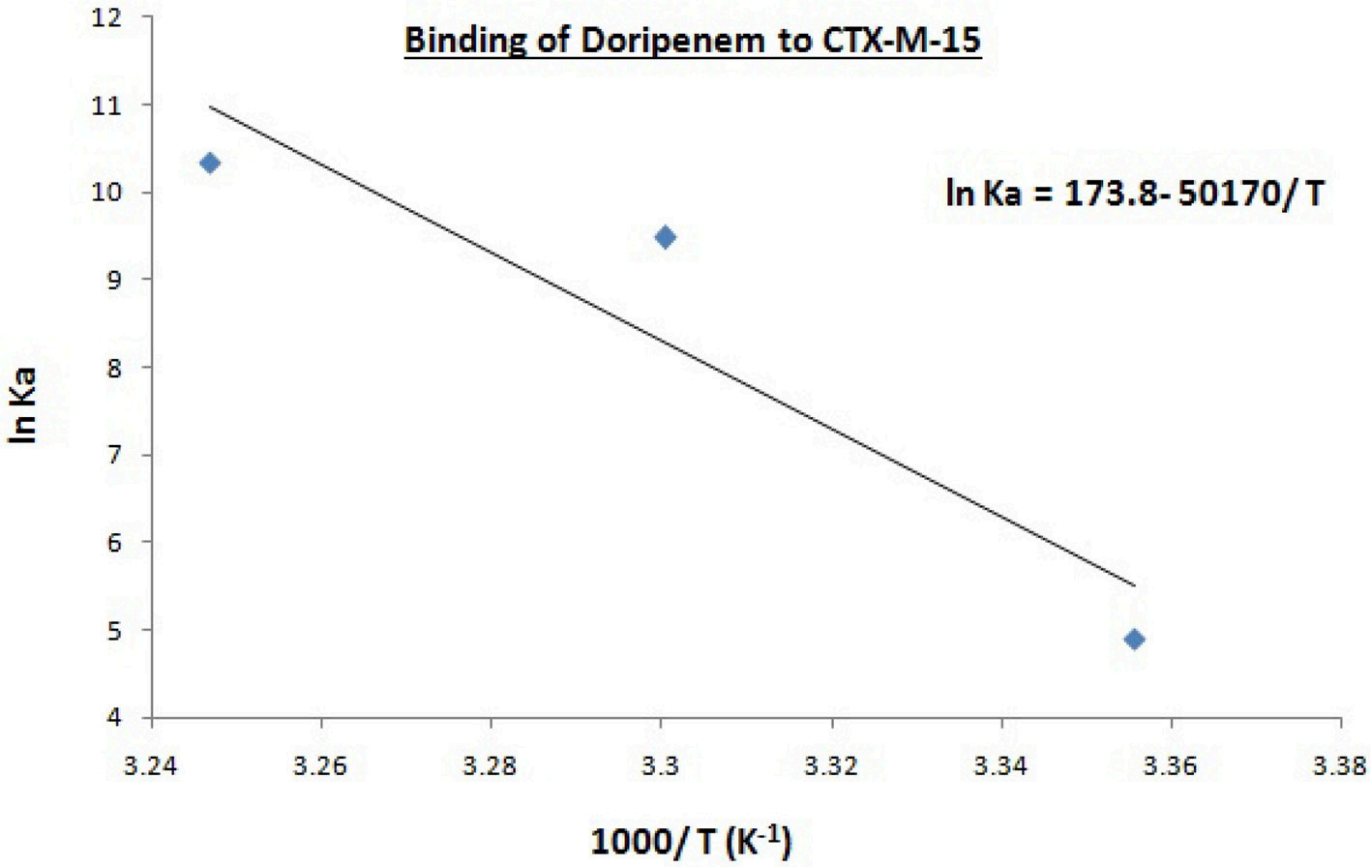
Van't Hoff plot for the binding of doripenem to 2 μM CTX-M-15 at 298, 303, and 308 K in the presence of doripenem (0 to 30 μM ) in 50 mM sodium phosphate buffer at pH 7.4 is shown.

Hence, the values of K_SV_ (Stern volmer constant) and Ka (binding constant) which were determined for the interaction of CTX-M-15 with doripenem were found in the order of 10^4^ M^−1^ and in the range of 10^2^–10^4^ M^−1^, respectively, with maximum value at 308 K (Table 1). The number of binding sites (n) for doripenem interaction was calculated as 1, using Equation (3) (Table 1). The Kq values was determined as 10^13^ M^−1^s^−1^, using Equation (2), from the ratios of K_SV_/τ°. The linear dependence of binding constant (Ka) on 1/T was observed using Van't Hoff plot as shown in Figure 6. Using the following Van't Hoff and thermodynamic equations (Equations 4 and 5, respectively), thermodynamic parameters, ΔS (change in entropy), ΔH (change in enthalpy), and ΔG (change in free energy) were determined.

(4) $$\text{ln }ka=\frac{\Delta S}{R}-\frac{\Delta H}{RT}$$

(5) ΔG=ΔH−TΔS

The value of enthalpy change was obtained using the slope of the plot (−Δ*H*/*R*), the value of change in entropy was estimated using the intercept (Δ*S*/*R*) of the plot and the value of Gibbs free energy change (Δ*G*) was calculated using Equation (5) (Table 2). Thermodynamic parameters for doripenem and CTX-M-15 interaction were obtained as 0.417 kJmol^−1^ Δ*H*, 1.44 kJK^−1^mol^−1^ Δ*S* whereas, Δ*G* was found to be −12.153, −23.882, and −26.506 kJmol^−1^ at 298, 303, and 308 K, respectively (Table 2).

**Table 2:** Thermodynamic parameters for the binding of doripenem to CTX-M-15 as estimated from fluorescence quenching at different temperatures.

**Temperature (K)**	**ΔH (kJmol^−1^)**	**ΔS (kJ K^−1^mol^−1^)**	**TΔS (kJ mol^−1^)**	**ΔG (kJ mol^−1^)**
298	0.417 ± 2.5	1.44 ± 1.6	430.602 ± 3.4	−12.153 ± 2.3
303			437.826 ± 2.8	−23.882 ± 1.4
308			445.051 ± 2.1	−26.506 ± 0.9

**Figure 7:**
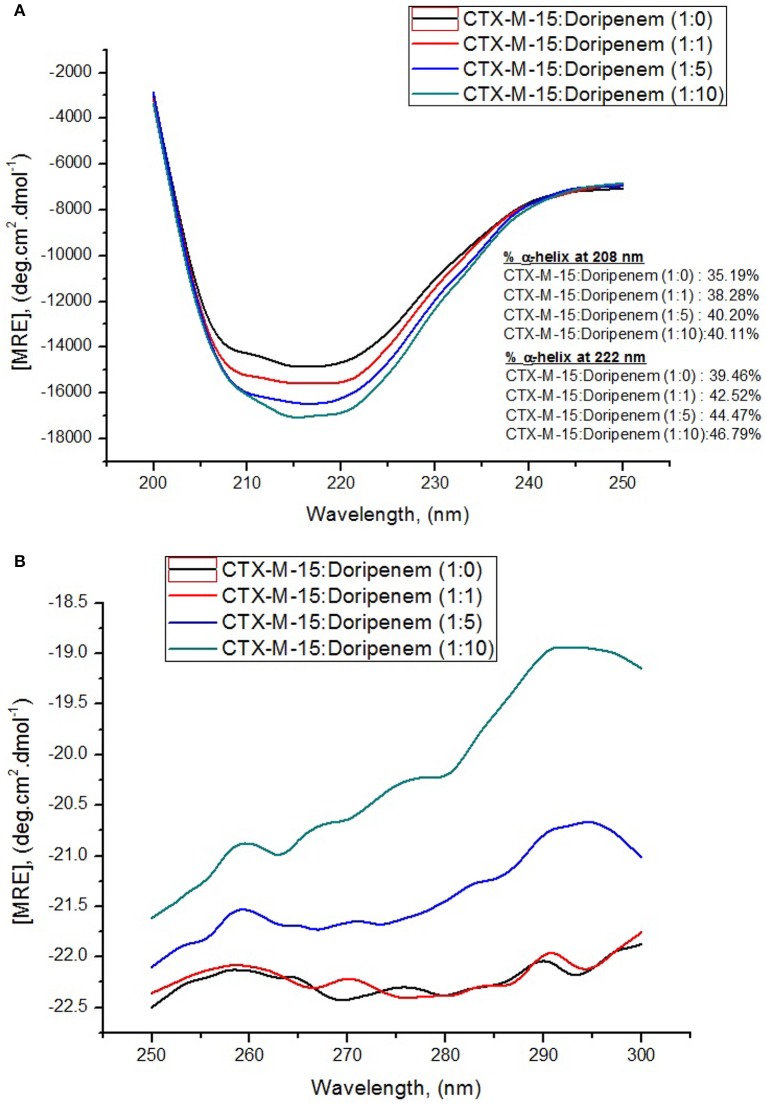
Panel **(A)** shows Far-UV CD spectral measurements and panel **(B)** shows Near-UV CD spectral measurements of 5 and 15 μM CTX-M-15 alone, respectively, along with doripenem in 1:1, 1:5, and 1:10 molar ratios in 50 mM Sodium phosphate buffer pH 7.4 at 298 K.

Circular dichroism was used for determining secondary structure changes, that is α-helices and β-sheets. Here, the effect of binding of doripenem on CTX-M-15 was monitored by UV-CD spectroscopy. Far UV-CD spectra was taken in the range of 200–250 nm and near UV-CD spectra was taken in 250–300 nm range. Figures 7A,B show the CD spectra of CTX-M-15 in far UV and near UV region, respectively, in the absence and in the presence of doripenem under different concentrations, at 298 K. In the far UV region, a characteristic α-helical protein curve of CTX-M-15 was observed in the absence of doripenem showing two negative bands at 208 and 222 nm which is further closely related to the far UV-CD spectra of CTX-M-1 (Perez-Llarena et al., 2011) and a positive peak at 278 nm in near UV region was closely resembling near UV-CD spectra of CTX-M-15 (Rehman et al., 2015). Calculation of mean residual ellipticity [MRE] in deg.cm^2^.dmol^−1^ from observed ellipticity was done using the following Equation (6) (Rehman et al., 2015).

(6) $$\text{MRE}=\frac{[\theta ]\text{obs}}{10\text{ncl}}$$

where [θ] obs stands for the observed ellipticity in mdeg, n is referred to the number of total amino acid residues present, which is 291 in the case of CTX-M-15 protein, c is the concentration of CTX-M-15 protein in moles, and l is the path length in cm. The percent content of α-helixes of CTX-M-15 protein in absence and presence of varying concentration of doripenem were calculated using the following equation from MRE values at 208 and 222 nm (Chen et al., 1972) and the resulting values are shown in Table 3.

(7) $$\%\alpha -\text{helix}=\left[\frac{[\text{MRE}]208\text{nm}-4000}{33000-4000}\right]\;*\;100$$

(8) $$\%\propto -\text{helix}=\left[\frac{\left[\text{MRE}\right]222\text{nm}-2340}{30300}\right]*100$$

It was observed that prior to doripenem treatment, values of MRE was found to be −14,206.7, −14,297.7, and −22.42 deg.cm^2^.dmol^−1^ at 208, 222, and 278 nm, respectively, and the amount of alpha helical content was 35.19 and 39.46% at 208 and 222 nm, respectively. In the presence of doripenem, in 1:1, 1:5, and 1:10 ratios, the alpha helical content of CTX-M-15 was increased to 38.28, 40.20, and 40.11% at 208 nm and to 42.52, 44.47, and 46.79% at 222 nm. Similarly at 278 nm, the MRE values in the presence of doripenem, in 1:1, 1:5, and 1:10 ratio were changed to −22.36, −21.60, and −20.20 deg.cm^2^.dmol^−1^, respectively.

**Table 3:** Spectral characteristics of CTX-M-15 alone and under doripenem binding condition.

	**MRE_208_ (deg.cm^2^.dmol^−1^)**	**% α helix at 208**	**MRE_222_ (deg.cm^2^.dmol^−1^)**	**% α helix at 222**	**MRE_278_ (deg.cm^2^.dmol^−1^)**
CTX-M-15+Doripenem (1:0)	−14, 206.7 ± 210	35.19 ± 1.9	−14,297.7 ± 112	39.46 ± 2.5	−22.42 ± 1.7
CTX-M-15+Doripenem (1:1)	−15, 103.8 ± 128	38.28 ± 2.1	−15.223.8 ± 142	42.52 ± 1.2	−22.36 ± 3.0
CTX-M-15+Doripenem (1:5)	−15, 658.1 ± 112	40.20 ± 3.0	−15,814.8 ± 103	44.47 ± 0.8	−21.60 ± 2.1
CTX-M-15+Doripenem (1:10)	−15, 632 ± 107	40.11 ± 1.0	−16,517.9 ± 92	46.79 ±1.6	−20.20 ± 0.5

**Figure 8:**
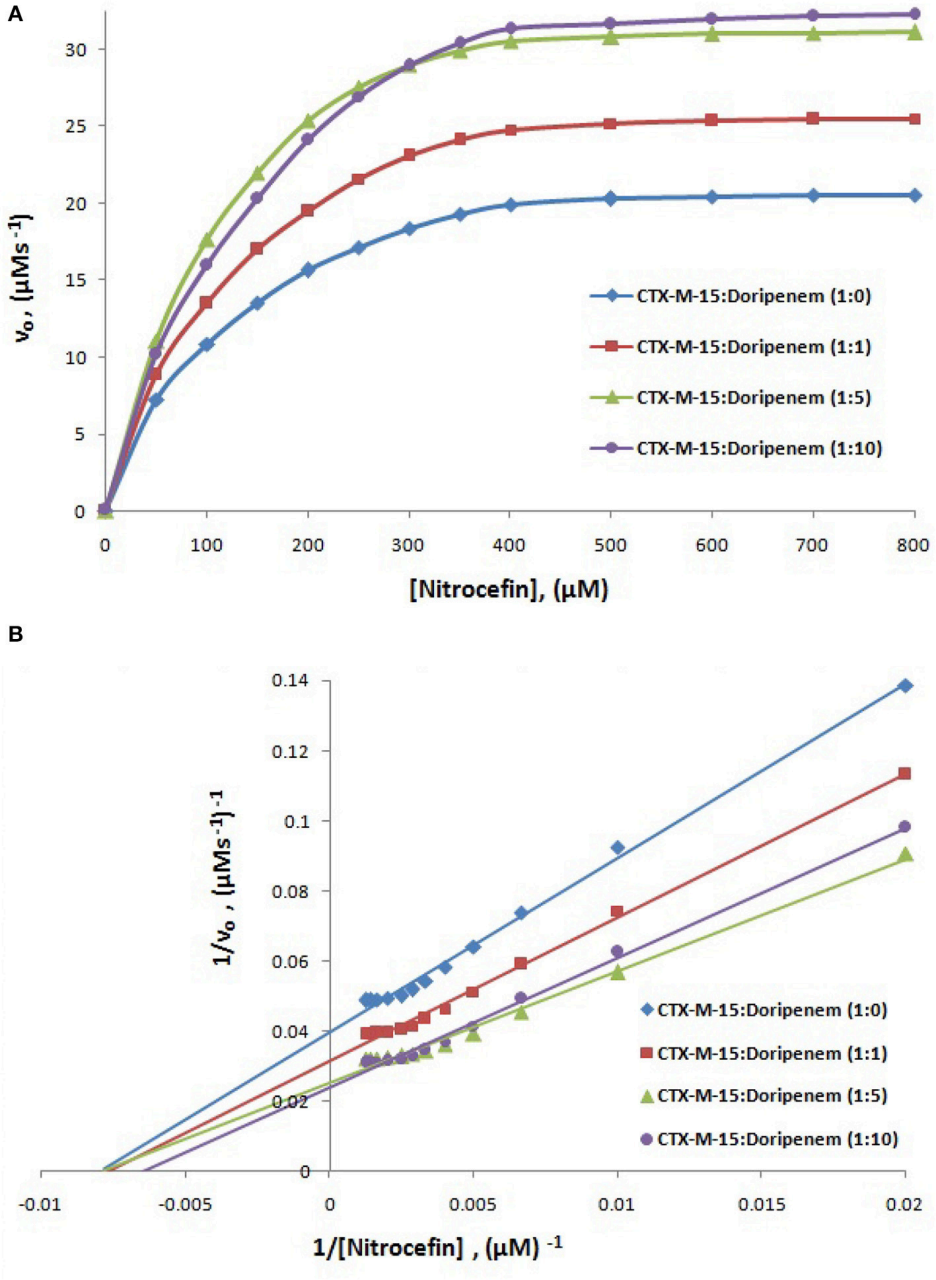
Panel **(A)** shows Michaelis-Menten plot and panel **(B)** shows Lineweaver-Burk plot of Steady-state enzyme kinetics study of Nitrocefin hydrolysis by CTX-M-15 (5 nM) in the absence and presence of 5, 25, and 50 nM of doripenem at 298 K in 50 mM sodium phosphate buffer, pH 7.4.

**Figure 9:**
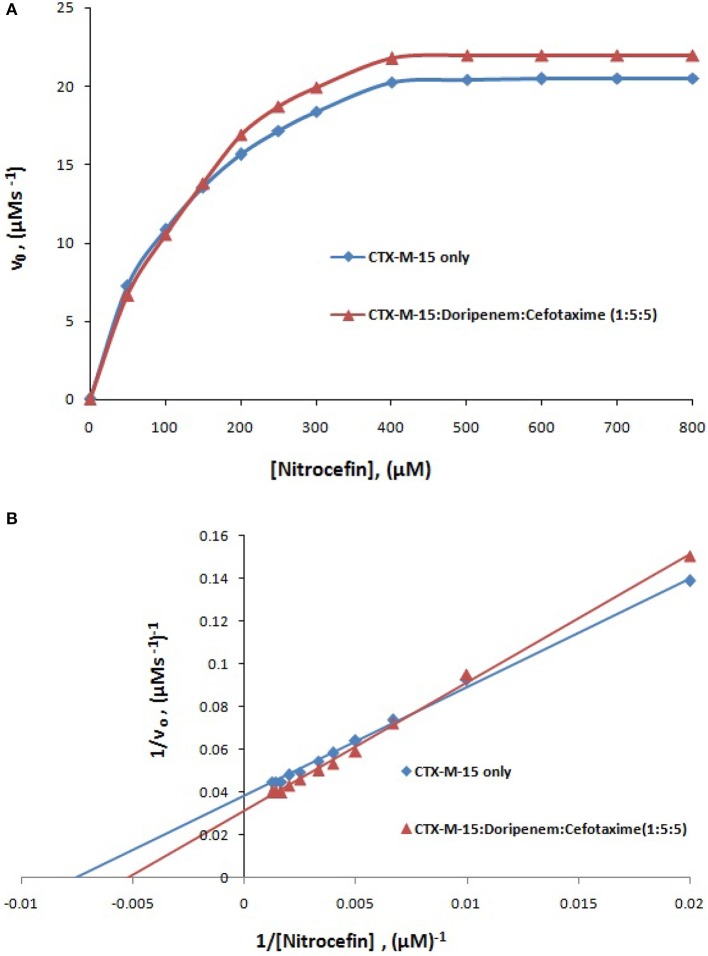
Panel **(A)** shows Michaelis-Menten plot and panel **(B)** shows Lineweaver-Burk plot of Steady-state enzyme kinetics study of nitrocefin hydrolysis by CTX-M-15 (5 nM) in the absence and presence of 25 nM each of doripenem and cefotaxime at 298 K in 50 mM sodium phosphate buffer, pH 7.4.

The steady-state kinetics of CTX-M-15 was carried out on nitrocefin to ascertain the effect of doripenem binding on the hydrolytic activity of enzyme. And the kinetic parameters, Kcat, Km, and Kcat/Km were determined using the following Michaelis–Menten equation:

(9) $$\text{v}=\frac{\text{Vmax}\left[\text{S}\right]}{\text{Km}+[\text{S}]}$$

(10) $$\text{Kcat}=\frac{\text{Vmax}}{\left[\text{E}\right]}$$

where v and v_max_ are the initial and maximum velocities of nitrocefin hydrolysis, respectively, [S] is the concentration of nitrocefin as substrate and [E] is the concentration of CTX-M-15 enzyme. The Michaelis–Menten plot and Line weaver-Burk plot are shown in Figures 8A,B and the kinetic parameters were deduced using line weaver-burk plot (Table 4). Increase in catalytic efficiency (Kcat/Km) of enzyme was found in the presence of doripenem, the value was increased from 40.155 μM^−1^s^−1^ to 48.947, 62.695, and 53.951 μM^−1^s^−1^ at 1:1, 1:5, and 1:10 molar ratios of CTX-M-15 and doripenem, respectively. Similarly increase in turnover number (Kcat) of enzyme was observed with the increasing concentration of doripenem. The values of kinetic affinity (Km) was also observed to be increased with the increase in concentration of doripenem. Moreover, to monitor the effect on rate of hydrolysis of CTX-M-15 in the presence of doripenem along with cefotaxime, another steady state kinetics experiment was carried out. The Michaelis-Menten plot and lineweaver-Burk plot thus obtained is shown in Figures 9A,B. The kinetic parameter Kcat/Km was observed to be decreased by 33.83 μM^−1^s^−1^, turnover number (Kcat) was increased to 6060.6 s^−1^ and Km was increased to 179.121 μM (Table 5).

**Table 4:** Steady-State kinetic parameters for hydrolysis activity of CTX-M-15 in presence of doripenem.

	**Km (μM)**	**Kcat (s^−1^)**	**Kcat/Km (μM^−1^s^−1^)**
CTX-M-15 only	127.71 ± 1.9	5128.2 ± 0.6	40.155
CTX-M-15+Doripenem (1:1)	131.806 ± 1.3	6451.6 ± 2.1	48.947
CTX-M-15+Doripenem (1:5)	127.6 ± 3.0	8000 ± 2.8	62.695
CTX-M-15+Doripenem (1:10)	154.458 ± 2.5	8333.2 ± 0.9	53.951

*Data are reported as average ± standard error from three independent experiments*.

**Table 5:** Steady-State kinetic parameters for hydrolysis activity of CTX-M-15 in presence of doripenem and cefotaxime.

	**Km (μM)**	**Kcat (s^−1^)**	**Kcat/Km (μM^−1^s^−1^)**
CTX-M-15 only	127.538 ± 1.8	5128.2 ± 0.6	40.209
CTX-M-15+Doripenem + Cefotaxime (1:5:5)	179.121 ± 1.6	6060.6 ± 1.1	33.830

*Data are reported as average ± standard error from three independent experiments*.

The FIC index was also determined for doripenem and cefotaxime in combination for BL21 (DE3) cells harboring *bla*_CTX−M−15_ gene, by checkerboard microdilution assay. The FICI value of doripenem and cefotaxime was found to be 0.12.

## Discussion

*bla*_CTX-M-15_ gene harboring *E. coli* BL21 cells showed susceptibility toward doripenem. At least 1189.05 nM concentration of doripenem is needed to inhibit bacterial cells. UV absorption spectroscopy showed change in spectral band position to longer wavelength with increase in molar ratios of doripenem. This shift in absorption peak is called as bathochromic shift or red shift. It occurs because of changes in the environmental conditions, happening around the protein and the drug doripenem, in increasing molar ratios, such as polarity and hydrophobicity/hydrophillicity (Dangkoob et al., 2015).

Mechanism of drug binding to enzyme can be explained by fluorescence quenching studies which measures decrease in the fluorescence intensity upon binding of drug (Eftink and Ghiron, 1976; Lakowicz, 1988). Quenching takes place because of several reasons such as due to reactions occurring in the excited state, transfer of energy from one form to another, molecular collision and due to formation of complexes. The phenomenon of quenching is dependent on pressure and temperature. Either static or dynamic quenching (Rehman et al., 2014), is dominating the process, since linear dependence of CTX-M-15 quenching was observed upon doripenem binding as obtained from spectra of fluorescence quenching studies. Values presented in Table 1 shows that significant interaction of CTX-M-15 and doripenem is taking place. A complex is formed between doripenem and CTX-M-15 as indicated by considerably high Kq values of the order of 10^13^ M^−1^s^−1^ with respect to maximum dynamic quenching constant (10^10^ M^−1^s^−1^). Greater interaction of doripenem with CTX-M-15 was observed at 308 K with respect to 298 and 303 K as shown by higher K_SV_ and Kq values. Lower K_SV_ and Kq values were observed at 303 and 298 K with respect to 308 K, signifying maximum stability at 308 K and initiation of quenching with ground state complex formation. Interpretation of obtained values of Ka and n showed that, CTX-M-15 has only one binding site for doripenem at all the three temperatures which may increase on increasing temperature, showing the microenvironment of binding site is becoming less hydrophobic. The binding constant (Ka) of doripenem was increased with the increasing temperature, most likely due to the integration of CTX-M-15-doripenem complex. Moreover, the negative values of ΔG indicated that the binding of doripenem to CTX-M-15 was a spontaneous process, the positive value of ΔH showed that the reaction was endothermic and the positive value of ΔS indicated that disorderness of the system is increased, as revealed by the analysis of thermodynamic parameters.

Structural changes occurring in CTX-M-15 protein upon doripenem binding was revealed by CD spectrometric study. It was observed that in far UV-CD range of 200–250 nm, two negative peaks of characteristic alpha helical protein were observed. At 208 nm, CTX-M-15 showed 8, 14.2, and 13.9% increase in alpha helix in 1:1, 1:5, and 1:10 molar ratios of CTX-M-15 and doripenem as shown in Figure 7A. At 222 nm, 7.7, 12.6, and 18.5% increase in the alpha helix was observed upon doripenem binding in 1, 5, and 10 molar ratio with respect to CTX-M-15. A characteristic peak of CTX-M-15 was observed in near UV-CD range of 250–300 nm, at 278 nm, showing 0.2, 3.6% increase and 9.9% decrease in MRE values at 1:1, 1:5, and 1:10 molar ratios of CTX-M-15 and doripenem, respectively (Figure 7B). Hence, CD spectra showed significant changes in the secondary structure of CTX-M-15 enzyme upon doripenem binding.

A chromogenic substrate, nitrocefin belonging to cephalosporin group of antibiotics is used to detect the presence of β-lactamase enzyme. It hydrolyses amide bond of nitrocefin, producing a shift in ultraviolet absorption which is eventually detected as a measure of β-lactamase activity (O'Callaghan et al., 1972). So in order to understand the effect of doripenem and doripenem in combination with cefotaxime binding on the efficiency of CTX-M-15, the CTX-M-15 enzyme kinetics study was carried out. The results showed that the affinity of CTX-M-15 toward nitrocefin was decreased in the presence of doripenem with the increase in concentration. Catalytic efficiency of enzyme to hydrolyse nitrocefin was increased by 21.8, 56.13, and 34.3% in the presence of doripenem in 1, 5, and 10 molar ratios, respectively, with reference to CTX-M-15. Maximum rise in the efficiency of enzyme was seen at 1:5 molar ratio of enzyme and doripenem. This enhanced efficiency of enzyme is probably due the structural changes in CTX-M-15, upon doripenem binding which make the enzyme more efficient to hydrolyse nitrocefin. In our earlier studies we have shown that presence of cefotaxime increases enzyme's efficiency to about 33% (Maryam and Khan, 2016). We asked if doripenem may reduce the efficiency of CTX-M-15 in presence of cefotaxime, hence, another enzyme kinetic study was performed and it was found to be decreased by 15.86%, when enzyme, doripenem, and cefotaxime was taken in 1:5:5 molar ratios. Hence, synergistic effect on CTX-M-15 hydrolysis with doripenem and cefotaxime at very low concentration (25 nM each) was established, where, efficiency was decreased by 48% as compared to cefotaxime treated CTX-M-15. It implies that doripenem induces structural changes which are probably responsible for decrease in cefotaxime hydrolysis by this enzyme.

A checkerboard microdilution assay was further performed to confirm the synergistic effect of doripenem and cefotaxime on the CTX-M-15 producing bacterial strain and It was found to be effective since doripenem in combination with cefotaxime showed FICI value of 0.12 (synergy is defined when FICI value is ≤0.5; White et al., 1996). Synergistic effect is observed when one drug increases the activity of another drug. Synergy requires atleast fourth-fold reduction in the MIC of both the antibiotics in combination as compared to each used alone (Rand et al., 1993). Therefore, microdilution assay showed that doripenem along with cefotaxime are able to inhibit the growth of bacterial cells at very low sub MIC concentrations.

## Conclusion

We conclude that doripenem at very low concentration of 25 nM, along with cefotaxime, reduces CTX-M-15 hydrolytic efficiency. Moreover, doripenem and cefotaxime show synergistic effect when taken in pair against CTX-M-15 producing bacterial strain. Study proves that combination of antibiotic therapy can be effective for treatment even if the bacteria are resistant to one of the antibiotics. Also during combination therapy, antibiotics administration in minimal concentration prevents the problems related to antibiotic overdose and its side effects. This study proposes the use of doripenem and cefotaxime in combination, at 25 nM concentration of each antibiotic, to inhibit the CTX-M-15 type β-lactamase enzyme. It further proves the use of reduced dose (below standard MIC) of antibiotics in combination against CTX-M-15 type β lactamase producing strains.

## Author contributions

LM performed experiments and wrote first draft of MS. AK design problem and guided the study and checked MS.

### Conflict of interest statement

The authors declare that the research was conducted in the absence of any commercial or financial relationships that could be construed as a potential conflict of interest.

## Abbreviations

IPTG: Isopropyl-β-D-1-thiogalactopyranoside
RFI: Relative fluorescence intensity
CD: Circular Dichroism
FICI: Fractional inhibitory concentration index.
