# Supplementary material for: Synergistic Effect of Doripenem and Cefotaxime to Inhibit CTX-M-15 Type β-Lactamases: Biophysical and Microbiological Views
Source: Front Pharmacol. 2017 Jul 5;8:449. doi: 10.3389/fphar.2017.00449 (PMC5496960; doi:10.3389/fphar.2017.00449)
Supplement: Supplementary file 1 [file Image1.PDF]

Figure S1:

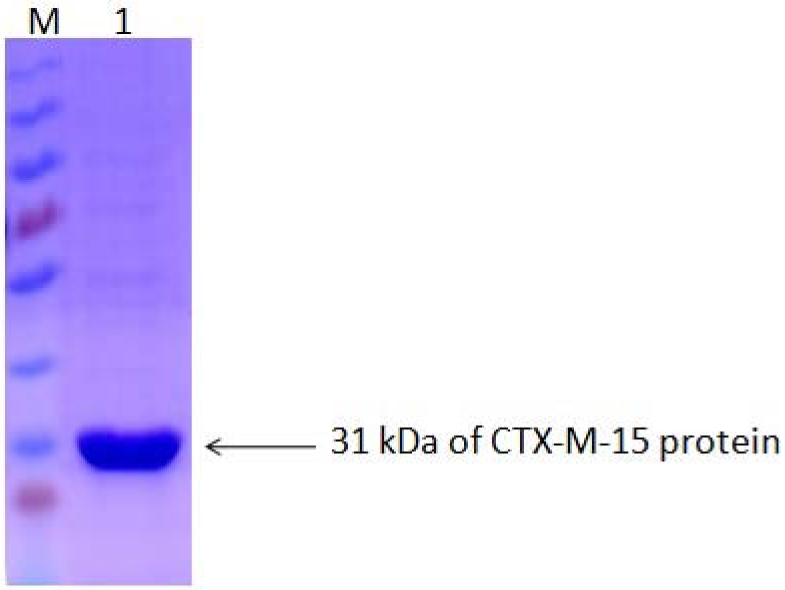

Figure S1: SDS polyacrylamide gel showing lane M of Prestained protein ladder and lane 1 of soluble protein fractions from *bla*$_{CTX-M-15}$ gene harbouring *E. coli* BL21 cell lysates, grown at 16 ^{o}C for 16 hours & induced with 0.2 mM IPTG.
